# Team climate and quality of care in primary health care: a review of studies using the Team Climate Inventory in the United Kingdom

**DOI:** 10.1186/1756-0500-2-222

**Published:** 2009-10-29

**Authors:** Teik T Goh, Martin P Eccles

**Affiliations:** 1 Institute of Health & Society, Newcastle University, 21 Claremont Place, Newcastle upon Tyne, NE2 4AA, UK; 2 Northumbria General Practice Vocational Training Scheme, Northern Deanery, 10/12 Framlington Place, Newcastle upon Tyne, NE2 4AB, UK

## Abstract

**Background:**

Attributes of teams could affect the quality of care delivered in primary care. The aim of this study was to systematically review studies conducted within the UK NHS primary care that have measured team climate using the Team Climate Inventory (TCI), and to describe, if reported, the relationship between the TCI and measures of quality of care.

**Findings:**

The databases MEDLINE, EMBASE, and CINAHL were searched. The reference lists of included article were checked and one relevant journal was hand-searched. Eight papers were included. Three studies used a random sample; the remaining five used convenience or purposive samples. Six studies were cross sectional surveys, whilst two were before and after studies. Four studies examined the relationship between team climate and quality of care. Only one study found a positive association between team climate and higher quality care in patients with diabetes, positive patient satisfaction and self-reported effectiveness.

**Conclusion:**

While the TCI has been used to measure team attributes in primary care settings in the UK it is difficult to generalise from these data. A small number of studies reported higher TCI scores being associated with only certain aspects of quality of care; reasons for the pattern of association are unclear. There are a number of methodological challenges to conducting such studies in routine service settings. Further research is needed in order to understand how to measure team functioning in relation to quality of care.

## Findings

There is considerable interest in what predicts, or results in, an increase in the quality of healthcare. Reviews of various empirical interventions that aim to improve the quality of healthcare have been inconclusive [1,2]. Authors have suggested the use of generalisable frameworks within which to consider issues relating to the quality of healthcare [3,4]. Ferlie and Shortell suggested that quality improvement in healthcare can be implemented at four different levels: individual professionals, groups and teams, organisations, and the overall system [5]. In a primary care setting the management of common chronic diseases is commonly provided by multidisciplinary teams of healthcare professionals and ancillary staff. The 'team' shares the responsibility for, and the provision of, care to patients [6-9]. Previous studies in the UK and elsewhere examined relationships between team working in primary care found that measurable aspects of team working were associated with improved outcomes such as effectiveness and patient satisfaction [8,10-13]. However, it remains uncertain which team attributes are important in improving the quality of care in primary care [7,13].

At the level of individual healthcare professionals there has been increasing use of "off the shelf" models or theories to measure a range of generalisable constructs that might predict higher quality care [14,15]. Within social and industrial psychology research one such team based measure that has the potential to inform the prediction of the quality of care is the Team Climate Inventory [16].

Team climate is defined as 'a team's shared perceptions of organisational policies, practices and procedures'[17]. Authors have suggested that shared team climate is a variable possessed by an organisation that can be measured and manipulated to change behaviour and improve the effectiveness of the organisation [18,19]. Given this, it is not unreasonable to assume that in healthcare organisations, greater effectiveness might result in improvements of quality of care that patients receive though causal mechanisms are not well articulated.

In the context of team innovation and performance, Anderson and West concluded that four group processes (facets) are important - team vision and objectives, participatory safety, task orientation, and support for innovation [17,20]. The Team Climate Inventory (TCI) was developed to measure facet specific climate for team innovation: (1) 'Team vision' measures the team members' perceived clarity, sharedness and attainability of the team objectives. (2) 'Participative safety' measures members' psychological safety and participation in information sharing and decision making. (3) 'Task orientation' measures members' reflection on appraisal, feedback and performance monitoring of work; and (4) 'Support for innovation' measures the perceived help in applying of new ideas and change [17]. Of these four domains, none seem to immediately translate directly into improvements of healthcare quality. However, a positive score on each would likely be a prerequisite for improved quality of healthcare. It is therefore likely that this is not a direct relationship, with a change in one of the constructs producing a measurable change in a health process or outcome measure, but, more likely, the effect is mediated via other aspects of individuals or organisations.

The original TCI (usually administered as a self report questionnaire) consists of 65 items (6 subscales) or 61 items (4 subscales), and various subsequent versions (44-item short form, 38-item, and a 14-item 'short version') have been developed[16,17,20-23] Responses for the items are either five or seven point Likert-type scales. Two indicators are generated from TCI responses: self-reported perceived team climate (mean score of the individual's responses to the four subscales) and the team's overall team climate (the mean of total team member scores).

## Aim

The aim of this study was to systematically review studies conducted within the UK NHS primary care that have measured team climate using the Team Climate Inventory (TCI), and to describe, if reported, the relationship between the TCI and measures of quality of care.

## Methods

### Inclusion criteria

Eligible studies were original research articles (of any design) that: measured team climate using any version of the TCI with primary care healthcare professionals (general practitioners, practice nurses, community or district nurses and relevant staff); and were conducted in either a primary care or education/training setting in the UK National Health Service (NHS).

### Measure of quality of care

Quality of care measures could include: physiological or biochemical measures used to indicate the management of chronic diseases; access, patient satisfaction, or self report team effectiveness.

### Search strategy

The databases searched were: MEDLINE (1988-December week 3 2007), EMBASE (1988-December 2007) and CINAHL (1982-December week 3 2007). We did not search the grey literature. Search terms used included "team climate", "teamwork$", "primary care", and Boolean operators (AND, OR). Given our interest in studies in the UK NHS, articles were limited to English language. We also checked reference lists of the included studies and hand searched the *Journal of Interprofessional Care*.

### Review methods

Studies retrieved by electronic searching were downloaded and screened by one reviewer (TG). Full text versions of all potentially relevant studies were obtained. The eligibility of each study was assessed and uncertainty whether to include those studies that did not meet inclusion criteria was resolved by discussion between the reviewers. For each study both reviewers abstracted descriptive data on the setting and participants and raw TCI scores and measures of quality of care if available. Where available, we abstracted the correlation coefficient (r) relating the team climate and the measure of quality of care. If this is not available, we abstracted the original results as presented in the studies.

## Results

### Identification of the studies

**Figure 1:**
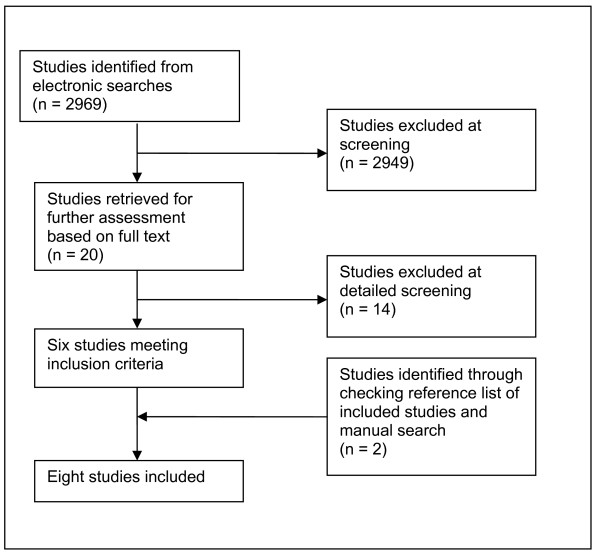
**Identification of studies included**.

Figure 1 shows the process of identification of included studies. Six studies were identified from the electronic searches [10,12,13,24-26] and a further two studies [27,28] were identified from checking the references of included studies and the hand searching.

### Included studies

The details of teams and individuals approached, responding and analysed are shown in Additional file 1. Overall, there were wide variations in terms of design and sampling of participants in the studies included. Three studies appeared to be based on the same overall sample (at two time points) and used stratified random sample of primary care teams [12,13,24]. The remaining five studies used convenience or purposive samples [10,25-28]. Across all eight studies from two to 68 practice teams were included.

All studies were observational, using questionnaire survey methods to elicit team climate [10,12,13,24-28]. Two studies used an evaluative design, albeit a weak one [27,28]. Of these two, one looked at changes in team climate before and after introduction of nurse-led minor illness service and a mental health clinic staffed by community psychiatry nurses [27], whilst the other conducted a before and after survey of a nursing team integration involving the core team of practice nurses, district nurses, and health visitors providing services mostly in shared premises [28].

### Teams and individual participants

Across the eight studies the number of teams approached per study ranged from two to 80 with responses reported from two to 68 teams (where reported, response rate 68 - 100%). The number of participants approached was not reported in three studies. When reported the number ranged from 85 to 1520 individuals, with responses reported from 40 to 720 (where reported, 41 - 100%). (See Additional file 1)

### Measurement of team climate and quality of care

Whilst all eight studies used the TCI questionnaire it is unclear whether the individual studies used the original 65 items version or the subsequent versions [16]. Four studies measured some aspects of quality of care including team effectiveness, while the remaining four either reported differences in team climate across different groups [25,26] or changes in team climate within the same group over time [27,28].

### Team climate in primary care teams

The results of the TCI scores are summarised in Additional file 2. Primary health care teams appeared to have lower team climate subscales scores when compared to other multidisciplinary professional teams [25,26]. Most studies reported the mean for TCI subscale scores [10,25-27], one reported scores for different professional subgroups [28], the remaining three did not report any TCI scores [12,13,24].

### Relationship between team climate and quality of care

Four studies examined the relationship between team climate and quality of care [10,12,13,24]. (See Additional file 3) Three (related) studies used three chronic clinical conditions and patient satisfaction surveys as their measures of quality [12,13,24]. The remaining study used a pre-developed self-report team effectiveness questionnaire [10].

Of the three related studies, two studies reported, from analyses of data from 42 practices, that higher team climate was associated with better access (measured using the General Practice Assessment Survey (GPAS) 53-item patient self-report questionnaire [12]), continuity of care, higher quality in the management of patients with diabetes, and higher scores for patient satisfaction (measured using the GPAS [12,24]). The multivariate regression analysis was performed on data collected in 1998 [12,24]. The details of quality assessment criteria were reported elsewhere [29,30]. However, these earlier results were not replicated in a subsequent study based in a sub-sample of the same practices and with updated measurement of TCI and clinical data [13].

## Discussion

There is a small body of literature describing the use of the TCI in primary care in the UK NHS. This review found only eight studies examining team climate across more than 200 teams in primary care. Only four studies examined the relationship between team climate and quality of care and four studies described team climate in various teams.

Whilst the TCI seems to offer a useful "off the shelf" measure of team attributes the identified literature does not offer a coherent view of the value of the instrument in the context of the UK NHS. Whilst it is worth remembering that it was never designed to predict quality of care its constituent domains (particularly innovativeness) make the exploration of its relationship with quality of healthcare delivered an appropriate area to explore. Unfortunately the identified studies are not particularly informative. Primary health care teams were shown to have lower scores when compared to teams from other organisational settings [25]. Compared to multidisciplinary team, uni-disciplinary teams were found to be more task-focused and supportive [26]. Amongst larger practices, GPs were found to have lower response rates [25].

The relationship between team climate and quality of care remains unclear although there were suggestions that higher TCI scores were related to better diabetes management, access, patient satisfaction and self-reported team effectiveness [12,13]. The precise nature of a relationship between the domains of the TCI and quality of care has not been well articulated. Given the interest in quality of care and team working the paucity of studies is surprising. The TCI is a widely validated questionnaire and has shown it can be used in primary care settings. Whilst using the self report TCI seems relatively economical and easy the time required to complete either the original 65-item or the revised 44-item questionnaire could lower the response rate. Linked to this, the need to have a minimum proportion of team members responding may also restrict the analysable data gathered; three of the reviewed studies found this an issue [12,13,24].

There are a number of limitations of this review. Our focus on the setting of the UK NHS primary care limits its generalisability to other settings. We only used one reviewer to screen the titles and abstracts before data extraction. There were a number of limitations of the studies included. As the authors of a number of the studies identified, the teams included in the studies might not be representative; those responding could have been the more motivated members of better functioning teams. The quality of the included studies varied. It was difficult to judge the internal validity of all of the reviewed studies. Some did not report the process of recruitment and analysis in sufficient detail to allow a judgement about risk of bias to be made. Those studies that used convenience or purposive samples will have limited external validity. There was considerable variation and discrepancy in how the studies reported their findings. From the perspective of a reviewer, it would be helpful if there was an agreed format if presenting results of such studies.

## Conclusion

This systematic review shows that, while the TCI has been used to measure team attributes in primary care settings in the UK it is difficult to generalise from these data. The relationship between TCI scores and quality of care is unclear. A small number of studies reported higher TCI scores being associated with only certain aspects of quality of care; reasons for the pattern of association are unclear. There are a number of methodological challenges to conducting such studies in routine service settings. Further research is needed in order to understand how to measure team functioning in relation to quality of care.

## Competing interests

The authors declare that they have no competing interests.

## Authors' contributions

MPE generated the idea for the study. TG carried out the literature search, data abstracting and analyses with input from MPE. TG wrote the first draft and both authors contributed to the subsequent versions. Both authors have read and approved the final manuscript. MPE will act as guarantor.

## Supplementary Material

Additional file 1 **Summary of study included: Description, sample and measures**. The file presents summary of study design, sampling method, data collection, response rates, and outcome measures. Click here for file

Additional file 2 **Summary of included studies: Analysis and results on team climate**. The file presents the results of studies that reported team climate scores. Click here for file

Additional file 3 **Summary of included studies: Analysis and results on team climate and quality of care**. The file presents the method(s) of analysis and results of studies examined the relationship between team climate and quality of care. Click here for file
