# Supplementary material for: Team climate and quality of care in primary health care: a review of studies using the Team Climate Inventory in the United Kingdom
Source: BMC Res Notes. 2009 Oct 29;2:222. doi: 10.1186/1756-0500-2-222 (PMC2775031; doi:10.1186/1756-0500-2-222)
Supplement: Additional file 1 — Summary of study included: Description, sample and measures. The file presents summary of study design, sampling method, data collection, response rates, and outcome measures. [file 1756-0500-2-222-S1.doc]

## Additional file 1

**Summary of study included: Description, sample and measures**

| **Study** | 1. **Study design** 2. **Sampling method** 3. **Data collection** | 1. **Team: a. Approached; b. Responded; c. Analysed** 2. **Participants : a. Approached;**   **b. Responded; c. Analysed** | **1. Number of TCI items**  **2. Item scale** | **Study outcome measures (method)**  **1. Clinical indicators**  **2. Patient evaluation: a. Approached; b. Responded (%);**  **c. Analysed**  **3. Team effectiveness**  **4. Other measures** |
| --- | --- | --- | --- | --- |
| West & Poulton 1997 | 1. Non-experimental  2. Convenience sampling of primary care teams attending team building workshops across the UK  3. Postal survey | 1, Teams: a. 80; b. NA ; c. 68  2. Participants: a. 1520; b. 745 (49%); c. 720  48 out of 68 primary care teams had less than 25% responses from GPs. | Number of items  Item scale | 1. NA  2. NA  3. NA  4. Comparison of team climate across multidisciplinary teams  from five different organisational settings |
| Poulton & West 1999 | 1. Cross-sectional  2. Convenience sampling of primary care teams attending team building workshops across the UK  3. Postal survey | 1. Teams: a. 68; b. 48; c. 46  2. Participants: 528; b. responded cal; c. analysed  GP 106; PN 56; DN 44; PM 42; Receptionist: 118; HV 63; Others 99 (midwives etc) | Number of Items  Item scale | 1. NA  2. NA  3. NA  4. Team Effectiveness (self-report) developed by Poulton & West (1993;1994), 25 dimensions grouped in four main factors: patient centred care, health care practice, teamwork, and efficiency. Scores were aggregated into overall effectiveness.  46 out of 68 teams (68%) returned effectiveness questionnaires. |
| Williams & Laungani 1999 | 1. Cross-sectional  2. Convenience sampling of teams attending team building workshops  3. Postal survey | 1. Teams: a. 30; b. 30 (100%); c. 30  19 multidisciplinary teams; 6 general practice teams; 2 single disciplinary teams; 3 NHS management teams  100% teams approached participated.  2. Participants: a. 259; b. 259 (100%);  HV: 51; Allied Health: 14; Managers: 21; SN: 22; Admin: 47; DN: 61; Other: 43 | Number of items  TCI self report | 1. NA  2. NA  3, NA  4. TCI scores |
| Haynes et al 2000 | 1. Controlled before and after study  2. Purposive sampling of a general practice with matched control practice  3. Postal Survey | 1. Team: a. 2; b. 2 (100%); c. 2  A 7-partner urban general practice with a matched practice.  2. Participants: a. NA; b. Response rate: Intervention 61-92%  Control: 41-65%  c. Number analysed not reported | Number of items | 1. NA  2. NA  3. NA  4. Measurements of mean TCI scores 8 months before and three times for 2 years after changes introduced  The changes were introduction of nurse-led minor illness  service and a clinic for mental health problems staffed by CPN |
| Ross et al 2000 | 1. Before and after study  2. Teams from two Health Authorities on implementing integrated nurse teams  Integration involved the core team of PN, DN, and HV working towards self managed, responsive, needs based service, mostly in shared premises. | Health Authority 1 – intervention introduced before study:  1. Teams: a. 8; b. Not reported; c. 4  2. Participants: a. 85; b. 44; c. Not reported  Only 4 of 8 practices returned enough data for team scores  Health Authority 2 – intervention planned and introduced over the study:  1. Teams: a. Not reported; b. 4; c. Not reported  2. Participants in year 1997  a. 57; b. 40; c. Not reported  GP 18, PN 10, DN 11, HV 5    Participants in year 1998  a. 57; b. 33; c. Not reported  GP 6; PN 9; DN 12; HV 6 | Primary Care Teamwork Questionnaire includes  TCI self report | 1. NA  2. NA  3, NA  4. Changes in mean TCI scores between two period of time |
| Campbell et al 2001 | 1. Observational study  2. Stratified random sample of general practices from six health authorities  3. Survey | Not reported | Not reported | 1. Chronic disease management for three conditions: asthma in adult, angina, and type 2 diabetes mellitus; preventive care: uptake cervical screening, primary childhood immunisation, MMR, and preschool vaccination  2. Patient evaluation: access and interpersonal care  3. Team effectiveness (details not reported)  4. Team climate |
| Bower et al 2003 | 1. Cross-sectional survey  2. Stratified random sample of general practices from six health authorities  3. Postal survey; chronic disease management data notes extraction; patient satisfaction, postal survey; Healthcare Team Effectiveness, postal survey. | 1. Teams: a. 75; 60 (80%); c. 42  2. Participants: a. 652; 387 (59%); c. Analysed cal  46 of 60 (77%) responded data for teamwork analysis. However, practices with a response rate less than 30% were removed leaving 42 practices for analysis.  Varied response rate mean 65% (SD=26%) from individual ractices. | Number of items  65  Item scale  5 item scale  7 item scale | 1. Chronic disease management (medical note) Items recorded past 14 months, past 5 yrs, and ever recorded for patient with clinical conditions: angina, asthma and type 2 diabetes 20 patients/practice  2. Patient evaluation of practice using General Practice Assessment Survey (GPAS) (self report) 53 items a. 11824; b. 4493 (38%); c. 3106  Not known reason why only 3106 of 4493 (69%) included in team analysis.  3. Health Care Team Effectiveness 21 items, three factors – professional practice, teamworking, patient centered care (one  additional factor was added – perceived team innovation) |
| Hann et al 2007 | 1. Non-experimental  2. Stratified random sample of general practices from six health authorities  3. Postal survey | Year 1998 dataset as above  Year 2007 dataset  Teams: a. 57; b. 42 (74%); c. 38  Participants: a. 736; b. 492 (67%); c. Not reported  Of the 60 practices in 1998 sample, 57 still exist in 2003, 42 (74%) agreed to participate. 4 of 42 practices were excluded due to low response rates <30%  Composition of participants not reported. | Number of TCI items=65  Item scale  5 item scale  7 item scale | 1. Chronic disease management (medical records) Items recorded past 14 months. past 5 yrs; and ever recorded for patient with clinical condition: CHD, Asthma, Type 2 Diabetes 1998: 20 patients/practice; 2003:12 patients/practice  2. Patient evaluation of practice (self-report) - General Practice Assessment Survey including items amended from General Practice Assessment Questionnaire Random sample: 200 patients from each practice  a. 8400 patients; b. (47%) (varied among practices)  3. Teams self-reported ratings of effectiveness (self-report)  4. Competing Values Framework for culture |

CPN = community psychiatric nurse; DN = district nurse; GP = general practitioner; HV = health visitor; PM = practice manager; PN = practice nurse
