# Supplementary material for: Team climate and quality of care in primary health care: a review of studies using the Team Climate Inventory in the United Kingdom
Source: BMC Res Notes. 2009 Oct 29;2:222. doi: 10.1186/1756-0500-2-222 (PMC2775031; doi:10.1186/1756-0500-2-222)
Supplement: Additional file 2 — Summary of included studies: Analysis and results on team climate. The file presents the results of studies that reported team climate scores. [file 1756-0500-2-222-S2.doc]

**Additional file 2**

**Summary of included studies: Analysis and results on** team climate

| **Study** | **Analysis** | **Item** | **Correlation** |
| --- | --- | --- | --- |
| West & Poulton 1997 | Standard one-way analyses of variance comparing scores across five samples of work groups | Mean scores for four subscales of TCI | TCI scores of members of team from five different organisational setting (n)   | TCI measure | PHC  (270) | Social  (360) | CMHT  (18) | Oil Co  (120) | NHS  (155) | F* | df | | --- | --- | --- | --- | --- | --- | --- | --- | | Objective | 4.87 | 5.12 | 5.24 | 5.34 | 5.34 | 22.4 | 4,1751 | | Participation | 3.55 | 3.64 | 3.83 | 3.84 | 3.59 | 19.1 | 4,1756 | | Task orientation | 4.45 | 4.78 | 5.05 | 4.75 | 4.61 | 11.2 | 4,1757 | | Support for innovation | 3.48 | 3.57 | 3.63 | 5.09 | 4.81 | 351.6 | 4,1757 |   PHC=primary health care; Social=social services; CMHT=community mental health team; Oil Co=oil company; NHS=NHS management team; *all p<0.001 |
| Haynes et al 2000 | Comparing intervention and control practice – four facets of TCI  Practice 1 = intervention  Practice 2 = control | Four subscales of TCI  Mean scores | Higher team climate scores in the intervention practice  Mean teamwork scores in intervention and control practice   |  | Survey 1 | Survey 2 | Survey 3 | Survey 4 | | --- | --- | --- | --- | --- | | Participation  Practice 1  Practice 2 | 3.5  3.3 | 3.5  3.5 | 3.5  3.5 | 3.6  3.7 | | Support for innovation  Practice 1  Practice 2 | 3.5  3.2 | 3.5*  3.3* | 3.6  3.4 | 3.6  3.5 | | Team objectives  Practice 1  Practice 2 | 4.6  4.2 | 4.7*  4.3* | 4.7  4.5 | 4.6  4.7 | | Task orientation  Practice 1  Practice 2 | 4.6  3.7 | 4.3  4.2 | 4.5  4.3 | 4.8  4.5 |   *p<0.05 difference between intervention and control practices |
| Williams & Laungani 1999 | one-way Analysis of Variance ANOVA (SPSS) | TCI total scores for each team for each subscales | TCI subscale according to staff type   | TCI subscale | MDC  (n=19) | NHSM (n=3) | PHC  (n=6) | SDT  (n=2) | F | df | p | | --- | --- | --- | --- | --- | --- | --- | --- | | Participation | 40.24 | 47.83 | 34.73 | 45.87 | 4.31 | 29 | <0.05 | | Support for innovation | 24.60 | 29.19 | 22.30 | 28.75 | 2.94 | 29 | >0.05 | | Vision | 22.87 | 18.61 | 9.74 | 14.12 | 3.97 | 29 | <0.05 | | Task orientation | 19.9 | 22.93 | 17.83 | 25.12 | 2.57 | 29 | >0.05 |   MDC=multidisciplinary clinic; NHSM = NHS management team; PHC=primary health care; SDT = single discipline team; df = degree of freedom |
| Ross et al 2000 | Quantitative: changes in the mean TCI scores for each team | TCI mean scores | Mean scores for professional groups (n)   | TCI | Norm for comparison | GP (28) | DN (32) | PN (20) | HV (11) | | --- | --- | --- | --- | --- | --- | | Participation | 3.56 | 3.97 | 4.09 | 3.53 | 3.35 | | Support for innovation | 3.38 | 3.57 | 3.82 | 3.60 | 3.13 | | Team objectives | 3.56 | 3.84 | 3.84 | 3.35 | 3.20 | | Task orientation | 3.26 | 3.39 | 3.94 | 3.20 | 2.91 |   For HA2 there was change of TCI scores between periods before and after intervention implemented. |

GP=general practitioner; DN=district nurse; PN=practice nurse; HV=health visitor
