# Supplementary material for: Team climate and quality of care in primary health care: a review of studies using the Team Climate Inventory in the United Kingdom
Source: BMC Res Notes. 2009 Oct 29;2:222. doi: 10.1186/1756-0500-2-222 (PMC2775031; doi:10.1186/1756-0500-2-222)
Supplement: Additional file 3 — Summary of included studies: Analysis and results on team climate and quality of care. The file presents the method(s) of analysis and results of studies examined the relationship between team climate and quality of care. [file 1756-0500-2-222-S3.doc]

**Additional file 3**

**Summary of included studies: Analysis and results on team climate and quality of care**

| **Study** | **Analysis**  **TCI variable mean/total score used** | **Outcome predicted by team climate** | | |
| --- | --- | --- | --- | --- |
|  |  | **Item** | **Significant predictor** | **Regression coefficient**  **(p value)** |
| Poulton & West 1999 | Block entry multiple regression  Not reported ?mean scores for each subscales aggregated | Patient care  Team work  Health care practice  Organisational efficiency  Overall effectiveness | TCI variables  TCI variables  TCI variables  TCI variables  TCI variables | 0.13 (NS)  0.30 (NS)  0.17 (0.09)  0.14 (NS)  0.23 (0.03) |
| Campbell et al 2001 | Series of backwards stepwise regression models  Combined TCI subscales into single score to identify predictors of high quality care | Diabetes  Access  Continuity of care  Overall satisfaction | Practices with higher scores for team climate had higher scores  2.37 (adjusted odds ration 0.36 to 4.38)  1.23 (1.09 to 1.38)  1.33 (1.18 to 1.50)  1.11 (1.05 to 1.19) | p=0.021  p =0.001  p<0.001  p<0.001 |
| Bower et al 2003 | Multiple regression backward selection (STATA)  Scores from Individual team members were aggregated to provide a mean overall team climate score. | Overall satisfaction  (N=42) | TCI  Booking interval (10min) | 1.35 (0.005)  22.16 0.000) |
|  |  | Diabetes management  (N=42) | TCI  Booking interval  Number of staff | 2.12 (0.031)  9.70 (0.007)  0.54 (0.014) |
|  |  | Self report innovation  (N=40) | TCI  Training status | 0.10 (0.003)  0.24 (0.038) |
|  |  | Self-report effectiveness (N=40) | TCI  Single-handed | 0.25 (0.000)  -0.50 (0.001) |
| Hann et al 2007 | Linear regression  Dependent variables (condition-specific quality; patient questionnaire scores) at patient level  Independent variables at practice level. Bootstrap re-sampling (10000 replication) for some outcome measures p<0.1 (in view of sample size and non-normality) see note below  Effects of TCI were expressed as differences in quality score expected for a 10-point difference ub climate scores (out of 100). Thus for TCI analyses regression coefficient represents estimated change in each outcome for a 10-point difference in TCI score. | Angina | TCI clarity of objectives  TCI task orientation | 4.15 (0.09)  2.40 (0.23) |
|  |  | Asthma | TCI clarity of objectives  TCI task orientation | 5.22 (0.17)  5.15 (0.10) |
|  |  | Diabetes | TCI  TCI clarity of objectives  TCI task orientation | 3.98 (0.15)  1.60 (0.58)  3.78 (0.10) |
|  |  | Patient satisfaction | TCI  TCI participation  TCI teamworking  Culture | 2.65 (0.11)  2.03 (0.09)  2.38 (0.27)  1.51 (0.58) |
|  |  | Patient communication | TCI participation  TCI teamworking  Culture | 2.14 (0.07)  2.83 (0.18)  1.03 (0.70) |
|  |  | Patient continuity | TCI participation  TCI teamworking  Culture | 3.72 (0.02)  4.18 (0.16)  3.74 (0.32) |

Note: Bootstrap p value for outcome variable versus TCI score: Angina=0.13; Asthma=0.14; Diabetes=0.10; Patient questionnaire overall satisfaction=0.02; Patient questionnaire communication=0.01; Patient questionnaire continuity=0.01.
